# Carbimazole-Induced Jaundice in Thyrotoxicosis: A Case Report

**DOI:** 10.7759/cureus.15241

**Published:** 2021-05-25

**Authors:** Abdul Majeed Maliyakkal, Tarik A A Elhadd, Vamanjore A Naushad, Nabeel M Shaath, Khalifa L Farfar, Mustafa S Ahmed, Sahiba M Basheer

**Affiliations:** 1 Medicine, Hamad Medical Corporation, Doha, QAT; 2 Clinical Medicine, Weill Cornell Medicine-Qatar, Doha, QAT; 3 Clinical Department, College of Medicine, QU Health, Qatar University, Doha, QAT; 4 National Diabetes Centre, Hamad Medical Corporation, DOHA, QAT; 5 Internal Medicine, Hamad Medical Corporation, Doha, QAT; 6 Clinical Department, College of Medicine, Qatar University, Doha, QAT; 7 Cardiology, Malabar Institute of Medical Sciences, Calicut, India, Calicut, IND

**Keywords:** jaundice, hyperthyroidism, carbimazole, thionamide, lft and tft

## Abstract

Carbimazole is a commonly used antithyroid drug in thyrotoxicosis. It is generally well tolerated, and its side effects include allergic skin reactions, gastrointestinal upset, agranulocytosis, and hepatotoxicity. Hepatitis is a rare but serious side effect. Here we report a case of carbimazole-induced hepatitis with severe cholestasis that was managed by switching to propylthiouracil. Most of the literature recommends radioiodine or surgery as the definitive treatment for hyperthyroidism in thionamide-induced hepatitis rather than switching to other thionamide. However, substitution of one thionamide for another can be tried as we did in this case, without any increased risk of hepatotoxicity as the mechanism of liver injury differs in both groups.

A previously healthy 30-year-old lady who was diagnosed with thyrotoxicosis one month earlier that was treated with carbimazole 60 mg daily was admitted to the medical ward with yellowish discoloration of sclera, urine, and pruritus of one-week duration. Systemic examination was unremarkable except for icterus. Investigation showed hyperbilirubinemia and elevated liver enzymes. A probable diagnosis of carbimazole-induced cholestatic hepatitis was made and the drug was discontinued. Other causes of hepatitis and cholestasis were excluded. Attempts to arrange radioiodine or treat the patient surgically were not successful. She was continued on propranolol and later started on steroids and propylthiouracil. The patient’s liver function tests (LFTs) started improving gradually. On follow-up, LFTs normalized at four weeks and thyroid function tests (TFTs) showed signs of improvement. The patient was followed up for six months after discharge and was doing well clinically on follow-up; her repeat TFT and LFT were completely normal.

Carbimazole-induced hepatitis is exceedingly rare; however, it should be considered in patients with jaundice and thyrotoxicosis. Despite reports of cross-reactivity of the two available antithyroid drugs, switching from carbimazole to propylthiouracil and steroid therapy may be an option if other options of definitive therapy could not be arranged or are contraindicated.

## Introduction

Carbimazole is the commonly used antithyroid drug in thyrotoxicosis. It is generally well tolerated, and its side effects include allergic skin reactions, gastrointestinal upset, agranulocytosis, and hepatotoxicity. Hepatitis is a rare but serious side effect. Here we report a case of carbimazole-induced hepatitis with severe cholestasis that was managed by switching to propylthiouracil (PTU). Most of the literature recommends radioiodine or surgery as the definitive treatment for hyperthyroidism in thionamide-induced hepatitis rather than switching to other thionamide [1,2]. However, substitution of one thionamide for another can be tried as we did in this case, without any increased risk of hepatotoxicity as the mechanism of liver injury differs in both groups.

## Case presentation

A 30-year-old lady with no significant past medical history who was diagnosed to have thyrotoxicosis (Graves’ disease) one month prior to admission (she was on carbimazole 60 mg daily and propranolol 20 mg thrice daily) was admitted to the medical ward with the complaints of yellowish discoloration of sclera, urine, and generalized itching of one-week duration. She denied fever and abdominal pain. She was never an alcohol consumer and denied intake of any other medications recently. Systemic examination was unremarkable except for icterus. Investigation showed hyperbilirubinemia (total bilirubin of 208 µmol/L, conjugated 124 µmol/L) and elevated liver enzymes (alanine aminotransferase [ALT] of 224 U/L, aspartate aminotransferase [AST] of 163 U/L, and alkaline phosphatase [ALP] of 347 U/L) (Table 1).

**Table 1:** Serial laboratory results WBC, white blood cells; AST, aspartate aminotransferase; ALT, alanine aminotransferase; ALP, alkaline phosphatase; T4, thyroxine; T3, triiodothyronine; TSH, thyroid-stimulating hormone

Tests (normal range)	On admission	6th day	11th day	23rd day (on discharge)	2 months	6 months
WBC (4–10 × 10^9^/L)	6.00				8.5	6.6
Hemoglobin ( 12–15 gm/dL)	12.7				13.9	14.2
Platelets (150–400 × 10^3^/µL)	251				244	183
Total bilirubin (3.5–24 µmol/L)	208	445	415	87	18	19
Direct bilirubin ( 0–8.6 µmol/L)	124	>171	>171	44		
AST (0–30 U/L)	163	131	64	62	18	25
ALT (0–31 U/L)	224	169	87	84	15	26
ALP (45–129 U/L)	347	345	217	117	122	106
Free T4 ( 8.4–19.1 pmol/L)	22.4		50.0		8.7	15.7
Free T3 (3.8–6.0 pmol/L)	6.15		11.7			
TSH (0.4–5.3 mIU/L)	<0.01		<0.01		2.4	1.9

Other causes of hepatitis and cholestasis were excluded (viral profile and autoimmune serology were normal). Obstructive causes were excluded by ultrasound and MRI imaging. A probable diagnosis of carbimazole-induced cholestatic hepatitis was made and carbimazole was discontinued on the day of admission. She was continued on propranolol. Attempts to arrange radioactive iodine treatment or treat the patient surgically were not successful due to technical and logistical reasons. Her thyroid function tests (TFTs) continued to worsen and she started to have recurrence of symptoms of hyperthyroidism, while the liver function tests (LFTs) slowly started trending down (Table 1). Hence, we started her on steroids and PTU on day 12. Her LFTs continued to improve gradually, and the symptoms of hyperthyroidism began to resolve. She was discharged after three weeks, and upon discharge her LFTs were as follows: ALT of 84 U/L, AST of 62 U/L, ALP of 117 U/L, total bilirubin of 87 µmol/L, and direct bilirubin of 44 µmol/L. The patient was followed up for six months after discharge and was doing well clinically on follow-up; her repeat TFT and LFT were completely normal.

## Discussion

The treatment for Graves’ hyperthyroidism consists of both rapid amelioration of symptoms with a beta blocker and a definitive measure aimed at decreasing thyroid hormone production with antithyroid medications (thionamides), radioactive iodine ablation, or thyroid surgery. Antithyroid drugs are associated with a higher rate of relapse compared to radioactive iodine or surgery­ [3]. Carbimazole and PTU are the commonly used thionamides in many parts of the world (methimazole in the United States). Mild derangement in LFTs has been described in hyperthyroidism per se, but it is usually mild and normalizes with treatment [4]. Both carbimazole and PTU differ in their mechanisms of causation of liver damage. PTU has hepatocellular toxic effects. Literature review shows that liver biopsy in PTU-induced liver injury patients has revealed nonspecific hepatocellular necrosis. The pathological findings may range from early signs of hepatocellular inflammation and swelling to submassive hepatic necrosis depending on the severity of the disease [5-10]. Carbimazole and its active metabolite methimazole have been associated with cholestatic jaundice without hepatic necrosis on biopsy. Previous case reports revealed liver biopsy specimen showing intracanalicular cholestasis and some mononuclear cell infiltrate in the portal triad, consistent with drug toxicity; indications of an autoimmune or viral pathogenesis were absent [11]. Most patients recover on drug discontinuation like our patient. Literature review shows that the mean time of onset of jaundice after starting the thionamide is 36 days [12]. In our patient, jaundice appeared one month after starting carbimazole. The mechanism of cholestasis is not fully understood, but it is thought to be an allergic reaction and dose-independent [11].

The definitive treatment of hyperthyroidism in our case could either be radioiodine or thyroid surgery; however, attempt to arrange either was unsuccessful initially. Hence, the patient was started on PTU and she tolerated it well, thyroid and liver functions improved gradually, and she was found to be euthyroid on follow-up at two months. On searching the literature, there were only very few case reports published in the past that showed switching from one thionamide to another in case of drug-induced hepatotoxicity. This case substantiates the fact that it is safe to substitute one thionamide over another if other modes of definitive therapy are contraindicated or not available.

## Conclusions

Carbimazole-induced hepatitis is exceedingly rare; however, it should be considered in patients with jaundice and thyrotoxicosis. Despite reports of cross-reactivity of the two available antithyroid drugs, switching from carbimazole to PTU and steroid therapy may be an option if other options of definitive therapy could not be arranged or contraindicated.
